# The non-synonymous mutation in bovine *SPP1* gene influences carcass weight

**DOI:** 10.1016/j.heliyon.2019.e03006

**Published:** 2019-12-13

**Authors:** Hirokazu Matsumoto, Ryosuke Kohara, Makoto Sugi, Azumi Usui, Kenji Oyama, Hideyuki Mannen, Shinji Sasazaki

**Affiliations:** a Laboratory of Animal Genetics, Faculty of Agriculture, Tokai University, Kumamoto, 862-8652, Japan; b Food Resources Education and Research Center, Graduate School of Agricultural Science, Kobe University, Kasai, Hyogo, 675-2103, Japan; c Laboratory of Animal Breeding and Genetics, Graduate School of Agricultural Science, Kobe University, Kobe, 657-8501, Japan

**Keywords:** Genetics, Animal breeding, Cattle, DNA sequencing, Gene mutation, Promoter, Carcass weight, Japanese Black cattle, Muscle, SNP, SPP1

## Abstract

Meat quality in beef cattle is controlled by genetic factors. *SPP1* (*secreted phosphoprotein 1*) gene, coding a multifunctional cytokine with diverse biological functions, is the candidate gene influencing carcass traits. In this study, we tried to discover DNA polymorphisms associated with beef quality in bovine *SPP1* gene, so that two SNPs (single nucleotide polymorphisms) in the promoter region and one SNP in the CDS (coding sequence) region were identified. Although the formers were predicted to alter *SPP1* expression, they did not show any effects on the traits. On the contrary, statistical analysis revealed that g.58675C > T, a non-synonymous mutation from threonine to methionine in the conservative region, had a significant effect on carcass weight. Carcass weight of the animals with C/T allele (473.9 ± 6.0 kg) was significantly heavier than that of the C/C homozygotes (459.2 ± 2.8 kg). Because *SPP1* gene functions in skeletal muscle cells as a positive regulator, the non-synonymous mutation might influence muscle development and remodeling, resulting in increased carcass weight of the C/T animals. Our results indicate that the SNP can be applied as a DNA marker for the improvement of beef cattle.

## 1 Introduction

The Japanese Black cattle, one of the four Japanese beef breeds known as Wagyu, are the major beef breed in Japan. One of the characteristics of Japanese Black cattle is high marbling, contributing to positive effect on the eating quality of beef. The main breeding objective in Japanese Black cattle is improvement of carcass traits. Although Beef Marbling Standard is regarded as one of the important traits because it influences price, efforts are thriving to improve other carcass traits including carcass weight, meat color and fatty acid composition.

Genetic factors are known to influence on carcass traits (Ibeagha-Awemu et al., 2008), and, therefore, improvement of carcass traits by the genetical method will be useful. Some studies have reported DNA polymorphisms which influence beef quality. For example, Taniguchi et al. (2004) identified a non-synonymous mutation in *SCD* (*stearoyl-CoA desaturase*) gene and elucidated that this SNP (single nucleotide polymorphism) influences fatty acid composition of beef cattle. Another example is *NCAPG* (*Non-SMC Condensin I Complex, Subunit G*) gene. A non-synonymous mutation in this gene has been shown to affect carcass weight (Eberlein et al., 2009; Setoguchi et al., 2009). Carcass traits are quantitative traits and modified by numerous genes. Therefore, additional DNA markers in other genes will be required for rapid and efficient breed improvement.

In the previous study, gene expression patterns were compared between cattle with superior carcass traits and that with inferior carcass traits, resulting that 12 genes were identified as the candidate genes for additional DNA markers of carcass traits (Matsumoto et al., 2013a). The genes identified by this comparison included the genes which are already known to have effects on carcass traits, *FABP4* (*fatty acid-binding protein-4*), *IGF1* (*insulin-like growth factor-1*) and *LEP* (*leptin*) genes (Curi et al., 2005; Nkrumah et al., 2007; Hoashi et al., 2007; Reyna et al., 2010; Narukami et al., 2011; Orrù et al., 2011), but association between the other candidate genes by the expression analysis and carcass traits remains to be elucidated. Among these candidates, we analyzed *ELOVL5* (*Elongation of very long chain fatty acids 5*) gene and revealed that a SNP identified in the promoter region influences fat-related traits (Matsumoto et al., 2013b). We also identified two SNPs in the promoter region of *MMP14* (*matrix metallopeptidase 14*) gene which influence fat-related traits (Matsumoto et al., 2019).

Bovine *SPP1* (*secreted phosphoprotein 1*) gene, also known as *OPN* (*osteopontin*), is the other candidate for additional DNA markers of carcass traits. This gene possesses 8 exons covering 57,376 bp on BTA6. An encoded protein is a multifunctional cytokine with diverse functions including bone re-modeling, wound healing and apoptosis (Singh et al., 2018). Some previous studies have reported the effects of this gene on economic traits. An in/del in the promoter region (T_9_/T_10_) has been shown to influence growth traits (Allan et al., 2007; White et al., 2007). A SNP in intron 4 also has an effect on body weight (Pareek et al., 2008). These data suggest that DNA polymorphisms in *SPP1* gene can be applied as DNA markers for breeding of Japanese Black cattle. The aim of this study was to develop additional genetic markers associated with beef quality, so that DNA polymorphisms in bovine *SPP1* gene were explored and their associations with carcass traits were examined.

## 2 Materials and methods

### 2.1 Animals and traits

In this study, 484 Japanese Black cattle (324 steers and 160 heifers) from diverse areas in Japan for purposes of progeny testing of sires were analyzed. Genomic DNA samples were extracted from the *musculus trapezius* by standard phenol chloroform extraction. Among these cattle, eight cattle were tested for sequence comparison to search for DNA polymorphisms. Additional 22 cattle were tested to analyze the linkage relationship between c.-1121C > T and c.-1117G > A. These cattle were chosen considering ancestry to minimize their genetic relationship. For the association study, genotypes of all animals including 30 cattle for sequence comparison were analyzed. The average ages in months ±SD of this group at slaughter was 28.70 ± 1.27.

Carcass traits were measured by official graders of the Japan Meat Grading Association: dressed carcass weight (kg), rib-eye area (cm^2^), rib thickness (cm), subcutaneous fat thickness (cm), yield estimate and Beef Marbling Standard. Fatty acid composition of the meat was studied as previously described (Matsumoto et al., 2019).

The samples analyzed in this study were obtained from carcasses, not living beef cattle. Therefore, the approval of Animal Welfare and Ethics Board is not required.

### 2.2 Sequence comparison

The coding sequence (CDS) regions, exons 3–8, and the promoter region of *SPP1* gene were tested. The approximately 2 kb upstream of the translation initiation site was analyzed as the promoter region where *SPP1* promoter activity was confirmed by Dudemaine et al. (2014). We designed the primers based on GenBank sequence (AC_000163.1) with Oligo7 (Molecular Biology Insights, Vondelpark, CO). For PCR, Go-Taq® (Promega Corporation, Madison, WI) and EX-Taq® (Takara Bio, Siga, Japan) were used as DNA polymerase. EX-Taq® (Takara Bio) was applied to amplify the region including exons 3 and 4 and Go-Taq® (Promega Corporation) to amplify the rest. PCR was performed using the following conditions: 35 cycles at 95 °C for 30 s, annealing temperature for 30 s, and 72 °C for 30 s. To amplify the partial promoter region (-1004 ~ -57), extension procedure was performed for 60 s. The primer sequences and the PCR conditions are shown in Table 1.

**Table 1:** Oligonucleotide primers used for sequencing.

Target region	Primer (5’ → 3′)	Tm (°C)	Size (bp)
Promoter (-1863 ~ -990)	F: TATAGCAGTTATCAGATCCAT R: CAAGAACAGAAAACTTATACG	60	919
Promoter (-1004 ~ -57)	F: AAGAGTATAATGGTAAACACT R: TGGAATCTTTTCTATTTTATA	50	990
Promoter (-655 ~ +2)	F: ATATTTTCACCTCTGTATTTAG R: ACGTCCTTTTTAGTAATGGTA	55	657
Exons 3, 4	F: GAGATGGAAAATAGAGGTGGC R: AGCAGGCACACAATAAATACT	60	539
Exons 5, 6	F: TTATCACTTAGAGACCCCTGT R: GCCTGGATAATCAAAAGGTGA	62	997
Exon 7	F: CCCTGACACCCATTTTTCTGG R: TGGGCTCTAATCATAACCATCTGA	60	384
Exon 8	F: GGTGTGGAAGTTAGAAGGCATTA R: TGCTTTAATGTATCCTTTTCGTTTT	54	467

F: forward primer; R: reverse primer.

Purification of the PCR products were perfomed using Exo-sapIT (affimetrix, Cleveland, OH). Subsequent sequencing was carried out by BigDye® Terminator v3.1 Cycle Sequencing Kit (Thermo Fisher Scientific, Wilmington, DE) and ABI PRISM® 3100 Genetic Analyzer (Thermo Fisher Scientific). To compare the obtained sequences, ClustalW (Thompson et al., 1994; Chenna et al., 2003) was utilized. The effects on gene function and expression of the SNPs were predicted by EMBOSS Transeq (EMBL-EBI, Hinxton, UK), Visual Gene Developer (Jung and McDonald, 2011) and ALGGEN-PROMO (Messeguer et al., 2002; Farré et al., 2003). In predicting putative transcription factor binding sites, transcription factors which dissimilarity rate were more than 10 were excluded. The locus of each SNP is calculated by the distance in base pairs from the translation initiation site.

### 2.3 PCR-RFLP genotyping

The primer sequences to amplify the region including c.-1117G > A were 5′-ATCACAGGGGACTGGACTCTTCTCG-3′ and 5′-ACCTTCCCAATGAAATGAGGCAGCG-3’. A mismatch nucleotide (underline) was adopted to introduce a *Fnu*4HI (New England Biolabs, Ipswich, MA) recognition site for the fragment, because no suitable restriction enzyme was detected at the site. The primer sequences for g.58675C > T were 5′-GCATGACGCACCTAAGAAGACGA-3′ and 5′-TCAAGGCTATGGAATTCTTGGCTGA-3’. This PCR product was digested by *Nla*Ⅲ (New England Biolabs). PCR was performed with Go-Taq® (Promega Corporation) using the following conditions: 35 cycles at 95 °C for 30 s, annealing temperature for 30 s, and 72 °C for 30 s. Annealing temperatures for c.-1117G > A and g.58675C > T were 58 °C and 60 °C, respectively.

### 2.4 Association analysis

Least squares method in JMP 13.2 (SAS Institute Inc., Cary, NC) was applied to analyze all traits. Analysis of variance (ANOVA) was executed to detect significant factors within a model. The model considered the effects of genotypes of the SNPs, prefecture, year and sex, and the linear and quadratic regressions of age at slaughter. Effect of sires was not considered in the analytical model due to small number of progeny per sire (3.2 head on average). Differences between least squares means for genotypes within a gene were examined by Tukey-Kramer's honestly significant difference (HSD) test. The animals with T/T allele of g.58675C > T were excluded from this analysis due to the limited number (n = 3).

## 3 Results

### 3.1 Polymorphism search

**Figure 1:**
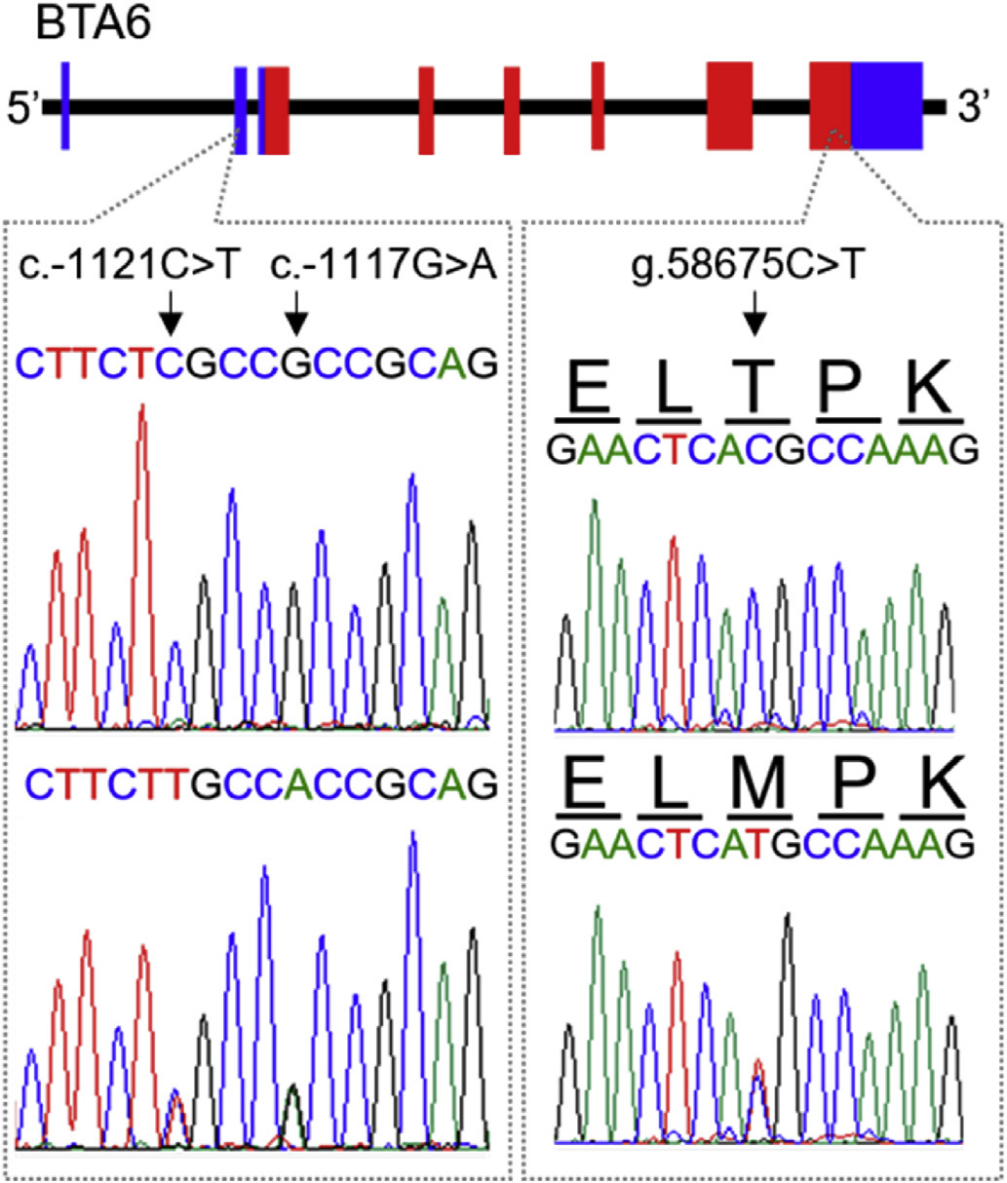
Identification of DNA polymorphisms in bovine SPP1 gene. Sequence comparison revealed two SNPs, c.-1121C > T and c.-1117G > A, in the promoter region and one non-synonymous mutation, g.58765C > T, in exon 8. g.58765C > T was predicted to cause amino acid substitution from threonine to methionine.

By sequence comparison with eight Japanese Black cattle, three SNPs in the *SPP1* gene were identified: c.-1121C > T (rs110329232) and c.-1117G > A (rs110254070) were identified in the promoter region and g.58675C > T (rs133929040) in exon 8 of the CDS (Figure 1). Two SNPs in the promoter region were identified in the adjacent area, so that we analyzed their relationship with additional 22 cattle. Animals with C allele of c.-1121C > T possessed G allele of c.-1117G > A and vice versa (data not shown). Because no exception was observed, these SNPs were suggested to be in complete linkage disequilibrium. The SNPs in the promoter region were identified in target sequence of transcription factors and predicted to influence their bindings (data not shown), suggesting that they might change expression pattern of the gene.

**Figure 2:**
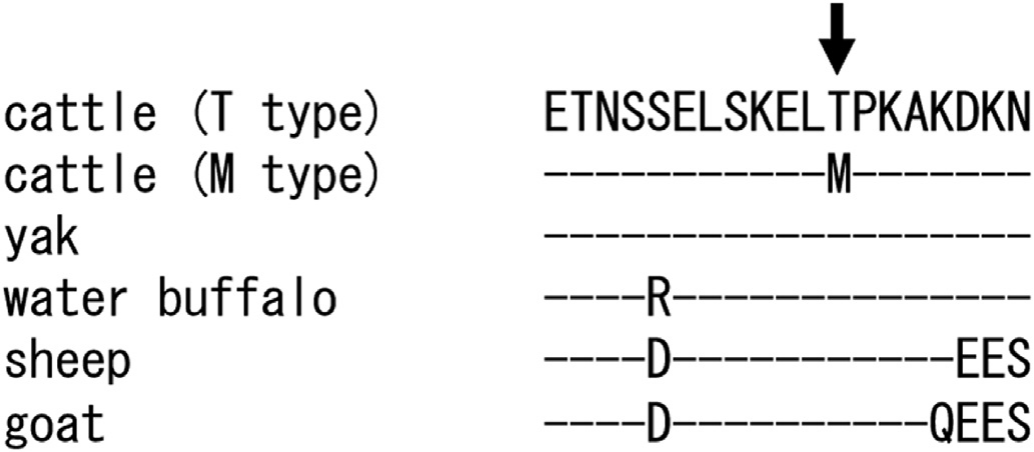
Homology study of SPP1 among Artiodactyla. g.58765C > T was predicted to influence on the 218th amino acid (arrow). The amino acid sequence on this region is highly conserved among Artiodactyla: yak (ELR54943), water buffalo (ABC02202.1), sheep (AAD38388.1) and goat (ABY21280.1). Dots indicate the same amino acids with above sequences.

The other SNP, g.58675C > T, was a non-synonymous mutation. This SNP was predicted to influence the 218th amino acid and to cause amino acid substitution from threonine, an amino acid with a polar uncharged side chain, to methionine, an amino acid with a hydrophobic side chain. Comparison of SPP1 amino acid sequences among cattle, yak, water buffalo, sheep and goat revealed that the region including this SNP is highly conserved among Artiodactyla (Figure 2). Because g.58675C > T was a non-conservative amino acid substitution in the conservative region, this SNP might alter the function and/or the structure of SPP1 protein. Meanwhile, this SNP was predicted not to disturb the mRNA second structure (data not shown), suggesting no influence on gene expression.

### 3.2 Associations between SNPs and traits

As influences of the SNPs on *SPP1* expression and function were suggested, genotypes of animals were analyzed for later association analysis. c.-1121C > T was excluded from this analysis, because this SNP was assumed to be linked with c.-1117G > A. The A allele of c.-1117G > A was minor and its frequency was 0.23. On the other hand, the minor allele of g.58675C > T was the T allele (0.09). The T/T homozygotes of g.58675C > T were rare in this population (n = 3). The ANOVA test was performed to analyze the SNPs’ effects on the traits (Table 2). The effect of g.58675C > T was observed on carcass weight (*p* = 0.0078), while c.-1117G > A did not show any effects on the traits. The HSD test revealed the detailed effects of g.58675C > T (Table 3). Carcass weight of the C/T cattle (473.9 ± 6.0 kg) was significantly heavier than those of the C/C cattle (459.2 ± 2.8 kg).

**Table 2:** Comparison of carcass traits and fatty acid composition between genotypes of the c.-1117G > A and g.58675C > T.

Trait	c.-1117G > A	g.58675C > T
Carcass traits		
Carcass weight	ns	**
Rib-eye area	ns	ns
Rib thickness	ns	ns
Subcutaneous fat thickness	ns	ns
Yield estimate	ns	ns
Beef marbling score	ns	ns
Fatty acid composition		
C14:0	ns	ns
C14:1	ns	ns
C16:0	ns	ns
C16:1	ns	ns
C18:0	ns	ns
C18:1	ns	ns
C18:2	ns	ns

*p < 0.05; **p < 0.01; ***p < 0.005; ns (non-significant).

**Table 3:** Effect of the g.58675C > T on carcass weight.

	g.58675C > T	
Trait	C/C (n = 407)	C/T (n = 83)
Carcass weight (kg)	459.2 ± 2.8^b^	473.9 ± 6.0^a^

Values are expressed as means with standard error by least squares estimates.

^a,b^ Means with different superscripts differ significantly at *p* < 0.05 (Tukey's HSD analysis).

## 4 Discussion

Three SNPs in *SPP1* gene were identified in the Japanese Black population. We expected effects of the SNPs in the promoter region because *SPP1* was among candidates associating with carcass traits identified by the microarray analysis (Matsumoto et al., 2013a), but they did not influence any carcass traits. On the other hand, the SNP in the CDS, g.58675C > T, showed an effect on carcass weight. As far as we know, this is the first report that this SNP influences economic traits of beef cattle, although Viale et al. (2017) reported the effect of the same SNP on milk coagulation properties of Holstein-Friesian cattle. This SNP was a non-synonymous mutation in highly conserved region, suggesting that the effects on carcass weight and milk coagulation properties might be derived from functional/structural alteration of SPP1 protein.

SPP1 protein undergoes various post-translational modifications to acquire its biological functions, including phosphorylation, glycosylation and proteolytic modification (Barry et al., 2000; Kazanecki et al., 2007; Christensen et al., 2008). The importance of phosphorylation toward SPP1 has been well characterized. For example, macrophage is activated by phosphorylated SPP1 (Weber et al., 2002). Phosphorylated form of SPP1 is also required for normal osteoclast function (Katayama et al., 1998). Meanwhile, SPP1 secreted by some types of cancers is likely less phosphorylated (Anborgh et al., 2011). The status of SPP1 phosphorylation can be different depending on cell types, indicating that alteration of phosphorylation might affect SPP1 function.

Threonine is one of the most commonly phosphorylated amino acids and phosphorylated sites are well conserved evolutionally in general. Therefore, the 218th threonine, the amino acid g.58675C > T was predicted to change into methionine, might be a phosphorylated site. Because proteolytic modifications are essential for SPP1 to be functional and some cleavage sites of this protein have been shown to be controlled by phosphorylation of specific amino acids (Sørensen et al., 1994; Christensen et al., 2005), the 218th threonine might be a target of proteolytic modifications and one of key amino acids for SPP1 function. In other words, g.58675C > T might alter SPP1 phosphorylation and its function as a signaling module, so that carcass weight might be affected. However, although Sørensen et al. (1994) identified most of phosphorylated sites in bovine SPP1 protein, it remains to be elucidated whether the 218th threonine is phosphorylated or not.

Many researchers have discussed the association between *SPP1* gene and muscle development and remodeling. SPP1 protein is highly expressed in muscle tissues of muscular dystrophy patients, although undetectable in normal muscle (Zanotti et al., 2011). A SNP in *SPP1* promoter has been shown to influence disease severity in Duchenne muscular dystrophy: rare allele of rs28357094 yields less SPP1 protein, causing greater phenotype (Pegoraro et al., 2011). The same SNP also modifies muscle size in healthy people. The effect was observed only in females, maybe because *SPP1* is an estrogen-sensitive gene (Hoffman et al., 2013). These phenomena might be derived from the function that *SPP1* plays in muscle regeneration (Kuraoka et al., 2016).

Recently, Nghiem et al. (2017) clarified a part of *SPP1* functions in skeletal muscle cells. They found that induction of SPP1 protein decreased myostatin (MSTN) expression and increased AKT serine/threonine kinase 1 (AKT1) expression and that MSTN expression was controlled by AKT1. Their findings suggest that SPP1 can decrease MSTN expression through AKT1 signaling. MSTN is a well-known and well-conserved negative regulator of skeletal muscle cell proliferation and differentiation (Carnac et al., 2007) and cattle lacking functional MSTN are known as “double muscled cattle” with increased muscle mass (Kambadur et al., 1997; McPherron and Lee, 1997). The non-synonymous mutation identified in this study might influence on this SPP1-AKT1-MSTN pathway: T allele of g.58675C > T might activate AKT1 stronger to suppress MSTN expression, resulting in increased carcass weight.

In the current study, we identified three SNPs in *SPP1* gene by sequence comparison. Statistical analysis revealed the association of one of the SNPs, g.58675C > T, with carcass weight by possibly influencing SPP1 function, structure and/or post-translational modifications. Our study indicates the possibility of *SPP1* g.58675C > T as an additional genetic marker to improve beef quality.

## Declarations

### Author contribution statement

Hirokazu Matsumoto: Conceived and designed the experiments; Wrote the paper.

Ryosuke Kohara, Makoto Sugi, Azumi Usui: Performed the experiments.

Kenji Oyama: Analyzed and interpreted the data.

Hideyuki Mannen, Shinji Sasazaki: Contributed reagents, materials, analysis tools or data.

### Funding statement

This work was supported by a Grant from The Ito Foundation (H30-#18).

### Competing interest statement

The authors declare no conflict of interest.

### Additional information

No additional information is available for this paper.
